# Editorial: New insights into the role of neuroinflammation and glial cells in the development of neurological disorders

**DOI:** 10.3389/fncel.2023.1170013

**Published:** 2023-03-14

**Authors:** Marta Pérez-González, Maite Solas, Yixing Wu, Virginia Mela

**Affiliations:** ^1^Department of Neuromuscular Diseases, UCL Institute of Neurology, University College London, London, United Kingdom; ^2^Department of Pharmacology and Toxicology, University of Navarra, Pamplona, Spain; ^3^IdISNA, Navarra Institute for Health Research, Pamplona, Spain; ^4^UK Dementia Research Institute at University College London, London, United Kingdom; ^5^Department of Medicine and Dermatology, Faculty of Medicine, University of Malaga, Malaga, Spain

**Keywords:** neuroinflammation, glial cell, microglia activation, astrogliosis, neurodegeneration

The largest cell fraction of the human nervous system is composed by glial cells. These non-neuronal cells are responsible for a myriad of functions in the brain including neurogenesis, synaptogenesis, neuronal excitability and synaptic connectivity, which support the maintenance of brain homeostasis. Under pathological circumstances, these cells play a pivotal role in neuroinflammation, a hallmark of several neurological and neurodegenerative disorders such as Parkinson's disease (PD), amyotrophic lateral sclerosis (ALS), multiple sclerosis, and Alzheimer's disease (AD). Therefore, unraveling the implication of non-neuronal cells in the development of these diseases and their contribution to neuroinflammation is a priority nowadays in neuroscience research.

Over the last decades, research has been focused on understanding the low-grade inflammation occurring during aging in the brain as a key player in various neurological disorders, giving special emphasis on neurodegenerative diseases. It is known that there is a cross-talk between different glial and peripheral immune cells in that complex and dynamic process which has consequences in neuronal function. Despite the huge amount of research investigating this specific topic, it is still unclear how these different cell-types interact with each other and how their responses contribute to the development or progression of a specific neurological disorder.

This special issue of Frontiers in Cellular Neuroscience entitled “*New insights into the role of neuroinflammation and glial cells in the development of neurological disorders*” compile four articles, among which three are original research and one is a review, going from AD to ALS always highlighting the central role of glial cells in those disorders.

The first original research article of this issue (Allendorf and Brown) pointed out to a specific lysosomal protein, neuraminidase 1 (Neu 1), as important player in the process of microglial reactivity during endotoxin/lipopolysaccharide infection. One of the main findings is the sensibilisation that neurons present to glutamate when Neu 1 is released after microglia reactivity, which could lead to neuronal death, proposing a new mechanism underlying neuroinflammation.

In the second manuscript (O'Neill et al.), the authors report the importance of sex dimorphism in microglial cells, especially in the development of AD. Using different approaches, ranging from the use of an animal model of AD pathology (APP/PS1 mice), to human brain tissue, they emphasized the distinct morphology of microglial cells between males and females. Microglia were more dystrophic in females, which could potentially explain the higher prevalence of AD. They also point toward iron as one important player in the process of microglial functional lost due to the correlation found between the iron-overload microglial cells in females, their dystrophic appearance, and changes in mitochondrial genes which lead to decreased mitochondrial functionality.

This special issue continues with a mini-review (Sanford and McEwan) focusing on the importance of Type I Interferon in different tauopathies. The authors compiled in this manuscript both protective and pathological responses of this cytokine in neurodegeneration-related tauopathies. The involvement of type I interferon pathways and its role in neuroinflammation have been studied more in AD. They extensively described the implication of Type I Interferon in tauopathies and how aging contributes to its signature. Another interesting section of this review focused on PD prion models.

In the last original research article of this special issue (Yamashita et al.), the authors describe a cell-type specific transcriptome of SOD1 mutant mice related to the development of amyotrophic lateral sclerosis (ALS). Considering astrocytic and microglial reactivity as the main players of the neuroinflammation orchestrated during ALS, the authors gave an extensive report of which genes are involved in the decrease of neurons and the increase of microglial and astrocytic reactivity. By using different enrichment analysis, they unraveled the database generated from the transcriptome of spinal cord tissue concluding that most of the differentially expressed genes in this mouse model are expressed predominantly in microglia. In addition to what has already been described in the literature, this manuscript sheds lights on potential therapeutic targets for SOD1-ALS, based on the combination of information coming from different cell types that are playing together an important role in the development of ALS.

In summary, the articles collected in the present special issue emphasize the essential role of glial cells in brain function and dysfunction, underlying the interest of these cells as potential future targets for the treatment of neurodegenerative diseases.

## Author contributions

VM wrote the manuscript. MP-G, MS, and YW edited the manuscript. All authors contributed to the article and approved the submitted version.

